# Histopathological, immunohistochemical, and ultrastructural evidence of spontaneous *Senecavirus A-*induced lesions at the choroid plexus of newborn piglets

**DOI:** 10.1038/s41598-017-16407-0

**Published:** 2017-11-29

**Authors:** Thalita E. S. Oliveira, Mariana M. Z. Michelazzo, Thiago Fernandes, Admilton G. de Oliveira, Raquel A. Leme, Alice F. Alfieri, Amauri A. Alfieri, Selwyn A. Headley

**Affiliations:** 1 Laboratory of Animal Pathology, Department of Veterinary Preventive Medicine, Universidade Estadual de Londrina, P.O. Box 10011, 86057-970 Londrina, Paraná Brazil; 2 Laboratory of Electron Microscopy and Microanalysis, Central of Multi-User Research Laboratories, Universidade Estadual de Londrina, Londrina, Paraná Brazil; 3 Laboratory of Microbial Biotechnology, Department of Microbiology, Universidade Estadual de Londrina, Londrina, Paraná Brazil; 4 Laboratory of Animal Virology, Department of Veterinary Preventive Medicine, Universidade Estadual de Londrina, Londrina, Paraná Brazil; 5 Molecular Biology Unit, Multi-User Animal Health Laboratory, Department of Veterinary Preventive Medicine, Universidade Estadual de Londrina, Londrina, Paraná Brazil; 6 Tissue Processing Unit, Multi-User Animal Health Laboratory, Department of Veterinary Preventive Medicine, Universidade Estadual de Londrina, Londrina, Paraná Brazil

## Abstract

Epidemic Transient Neonatal Losses (ETNL) is a disease of piglets caused by Senecavirus A (SVA) in which the method of dissemination and associated lesions are not well-defined. This study investigated the possible SVA-induced lesions by examining spontaneous infections in newborn piglets. Histopathology revealed ballooning degeneration of transitional epithelium, nonsuppurative meningoencephalitis, plexus choroiditis, and atrophic enteritis. RT-PCR identified SVA in all tissues evaluated and sequencing confirmed these results. Positive immunoreactivity to SVA was observed in endothelial and epithelial tissues of all organs evaluated. Semithin analysis revealed vacuolization of apical enterocytes of the small intestine, balloon degeneration and necrosis of endothelial cells of the choroid plexus (CP) and nonsuppurative choroid plexitis. Ultrathin evaluation demonstrated hydropic degeneration of apical enterocytes, degeneration and necrosis of endothelium of CP fenestrated capillaries, degeneration of ependymocytes associated with intralesional viral particles. It is proposed that SVA initially infects apical enterocytes of newborn piglets and probably enters the circulatory system with entry to the brain via the CP, by first producing an initial inflammatory reaction, with subsequent encephalitic dissemination. Consequently, SVA probably uses an enteric-neurological method of dissemination.

## Introduction

*Senecavirus A* (SVA), formerly known as Seneca Valley virus, is the only representative of the genus *Senecavirus*, family *Picornaviridae* ^1^. SVA is currently associated with the syndrome known as Epidemic Transient Neonatal Losses (ETNL), which is clinically manifested by lethargy, cutaneous hyperaemia, diarrhoea, neurological signs and/or sudden death in newborn piglets in pig farms from Brazil^2–5^, North American^4,6,7^, China^8,9^, Thailand^10^, and Colombia^11^. Furthermore, ETNL was reported as affecting piglets under 10 days of age^3^. Piglet mortality due to SVA in Brazil has been estimated as 20–30% in the states of Paraná and Santa Catarina^2^, and 30–70% in the states of Minas Gerais and Goiás^5^. Additionally, in Brazil the disease in piglets and sows occur simultaneously^2,5^, while in the USA, sows are affected initially causing vesicular lesions with subsequent newborn mortality occurring weeks thereafter^6^.

The detection of a pathogen in tissue by Polymerase Chain Reaction (PCR) and/or Reverse Transcription-Polymerase Chain Reaction (RT-PCR) in the absence of related pathological alterations does not necessarily imply that the identified agent is associated with a specific lesion or disease. Consequently, it is of extreme importance to identify the presence of the pathogen associated with histopathological lesions in affected tissues^4^. Immunohistochemistry (IHC) is an important diagnostic technique frequently used for the detection of a specific protein associated with the antigen of infectious disease agents in the microscopic lesions of formalin-fixed paraffin embedded (FFPE) tissues^12^. Another important technique that assists in the diagnosis of infectious diseases is *in situ* hybridization (ISH), due to the detection of specific nucleic acid (DNA, RNA, or messenger RNA) segments of an infectious agent in the sample under evaluation^13,14^.

Currently, investigations related to ETNL have been restricted to few studies^3,4,6^, that involved small populations of animals, varying from nine^6^ to ten^3,4^ necropsies of piglets affected by this syndrome. It must be highlighted that in one study 66.7% (6/9) of the brain samples evaluated were negative for SVA by ISH^4^, while 80% (8/10) of the piglets investigated by our group contained antigens of SVA in the choroid plexus (CP) by IHC, and in three of these SVA was amplified from the brain by RT-PCR^3^. We hypothesized that the elevated detection of SVA in the brain is probably associated with obtaining brain fragments containing the CP, and that this viral pathogen may have the ability to alter the integrity of vessels of the CP, since we have consistently identified antigens of SVA at the vascular endothelia of capillaries at this anatomic location^3^. Furthermore, since we had identified atrophic enteritis and histopathological evidence of lesions to the CP in piglets from a previous study^3^, we hypothesized that viral particles might have been present in the ballooning degeneration identified in cases of atrophic enteritis and at the CP of these diseased piglets. The CP is the location of the blood-cerebrospinal fluid barrier (BCSFB), one of the barriers within the brain that prevents the entry of infectious agents to the central nervous system^15–18^.

This study describes the gross, histopathological, IHC, and transmission electron microscopy (TEM) findings associated with spontaneous SVA-induced ETNL infections in 54 newborn piglets from Southern Brazil, validates the findings described in an earlier study by our group with a smaller population of piglets^3^, and provides initial evidence for the possible method of dissemination of SVA in newborn piglets.

## Results

### Clinical manifestations and gross findings

Of the 54 piglets with clinical manifestations suggestive of ETNL, in 80% (43/54) of these, at least one of the tissues/organs evaluated had positive immunolabelling for SVA by IHC and SVA RNA was identified by RT-PCR. Consequently, only the results of piglets with at least one tissue that was IHC positive for SVA were considered during this study. Diarrhoea was the most common clinical manifestation (91%; 39/43) observed in these piglets. The stomach of all piglets, including those with cachexia (9%; n = 4/43), were filled with milk; only 24% (13/54) of these piglets had neurological signs. The distribution of the age of the piglets is given in Supplemental Fig. 1; age was considered as the time of death. Consequently, most (81%; 35/43) piglets that died due to ETNL were between 2–5 days of age.

**Figure 1:**
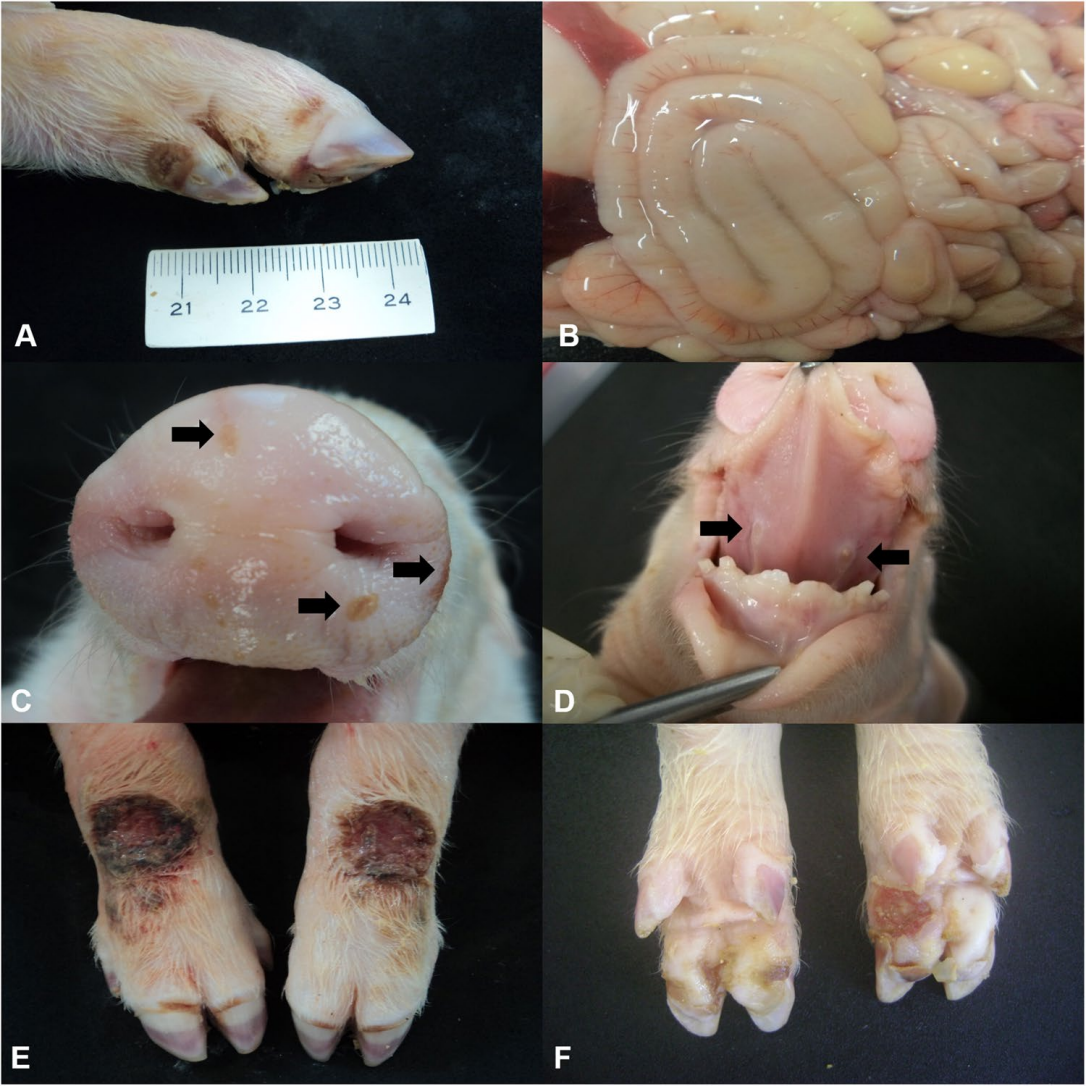
Gross findings observed in newborn piglets naturally infected by SVA. Coronary band, 4-day-old piglet; observe the erosive lesion (0.7 cm in diameter) at the coronary band of the right forelimb (**A**). Abdominal cavity, 3-day-old piglet; there is mesocolonic oedema; the intestinal segment is dilated by diarrhoea and the mesocolon is swollen (**B**). Snout, 4-day-old piglet; there is multifocal ulceration of the skin (arrow) of the snout after rupture of a vesicle (**C**). Tongue, 6- day-old piglet; there is symmetric ulcerative glossitis (arrow) at the ventral face of the tongue (**D**). Fore limbs and hooves, 7-day-old piglet; observe ulcerations and crusting lesions (2 cm diameter) at the coronary bands and metacarpus with an erosive lesion at the margin of the coronary band of the forelimbs (**E**). Palmar footpad, 3-day-old piglet; there is a large ulceration (1.2 cm diameter) at the right footpad (**F**). Scale in cm.

The most frequent gross manifestations observed during autopsy were liquid faeces (91%; 39/43), renal petechial haemorrhage (79%; 34/43), faint rib impressions on the pleural surface of the lungs (77%; 33/43), pulmonary oedema and congestion (60%; 26/43). In addition, there were cases of ulcerative lesion at the coronary band (35%; 15/43; Fig. 1A), mesocolonic oedema (32%; 14/43; Fig. 1B), vesicles at the snout (30%; 13/43; Fig. 1C), and lymphadenopathy (28%; 12/43). Less frequently occurring lesions included hyperplasia of Peyer’s patch (16%; 7/43), ulcerative glossitis (16%; 7/43; Fig. 1D), skin abrasion at the carpus (14%; 6/43; Fig. 1E), ulcerative gingivitis (14%; 6/43), ulcerative lesion of the hoof (12%; 5/43; Fig. 1F), and ulcerative cheilitis (9%; 4/43). Furthermore, there were concomitant vesicles at the muzzle with ulcerative lesions at the coronary band in 21% (9/43) of the piglets investigated.

### Histopathological findings associated with Senecavirus A observed in newborn piglets

**Figure 2:**
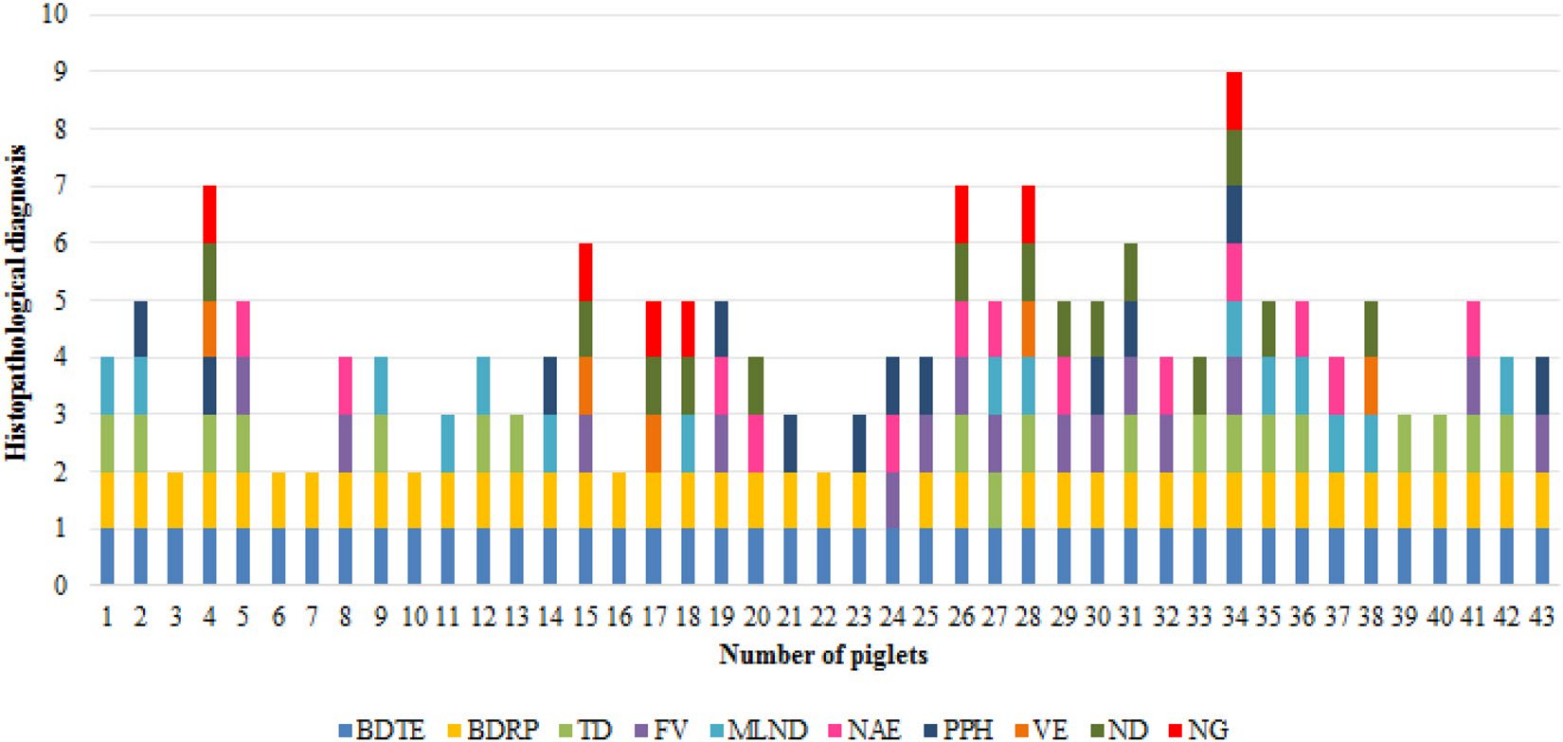
Graphical distribution of the principal histopathological findings in 43 piglets with SVA. Legend: BDTE, ballooning degeneration of transitional epithelium; ballooning degeneration of renal pelvis (BDRP), tonsilar depletion (TD), fusion of villi (FV), mesenteric lymph node depletion (MLND), necrosis of apical enterocytes (NAE), Peyer’s patch hyperplasia (PPH), vacuolization of enterocytes (VE), necrotizing dermatitis (ND), necrotizing glossitis (NG).

**Figure 3:**
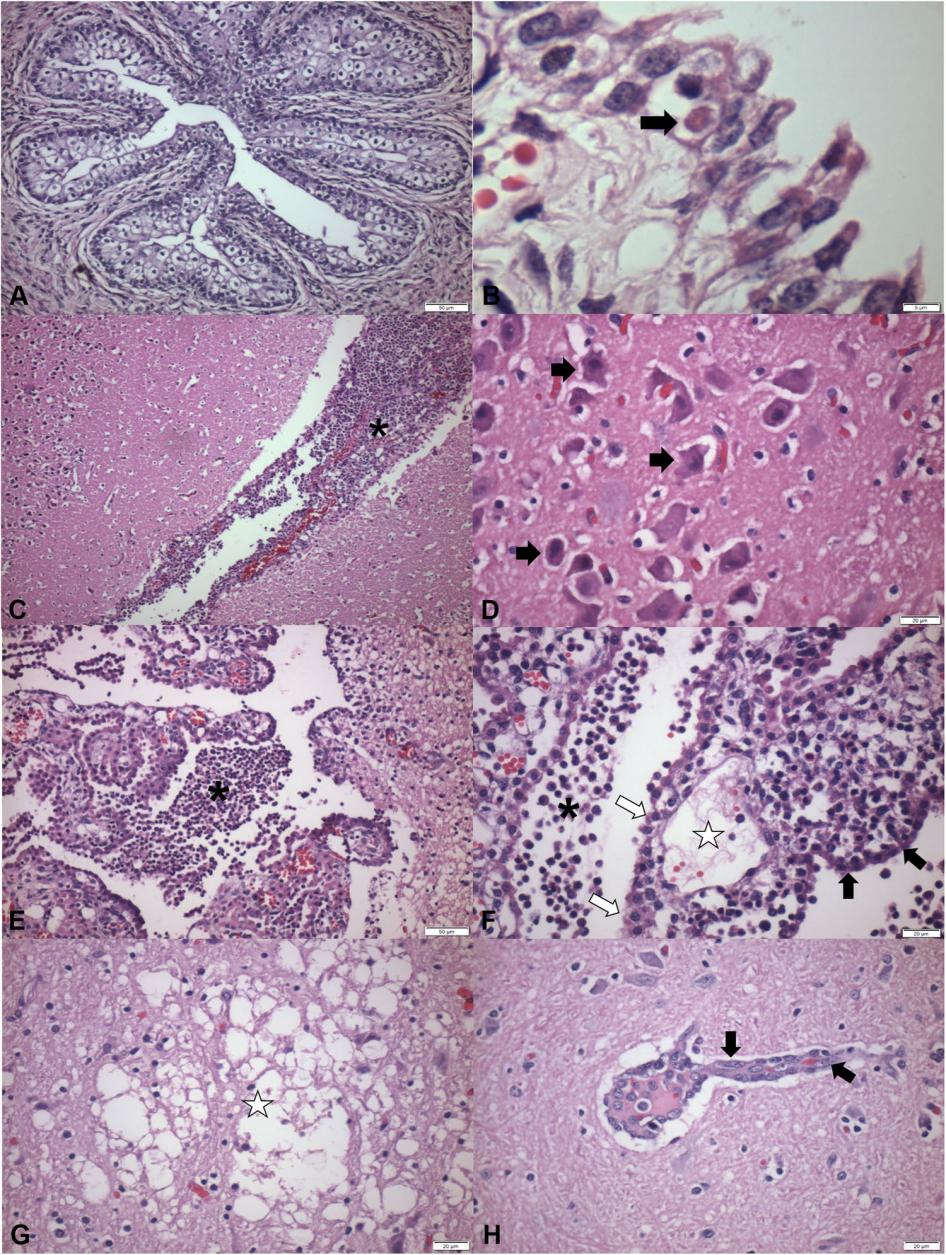
Histopathological findings observed in newborn piglets naturally infected by SVA. Ureter, there is severe hyperplasia and balloon degeneration of the urothelium (**A**). Urinary bladder, observe ballooning degeneration of the transitional epithelium associated with an intracytoplasmic, eosinophilic inclusion body (arrow) (**B**). There is severe focally extensive nonsuppurative meningoencephalitis (*, **C**) and cerebrocortical necrosis of the brain (**D**); observe several ischemic neurons (arrows). There is severe multifocal nonsuppurative choroid plexitis (*) of the lateral ventricle (**E**); observe the intraluminal accumulation of fibrin (★) in a fenestrated capillary, degeneration of ependymocytes and (open arrows), ballooning degeneration (arrow head) as compared with normal (closed arrow) ependymocytes (**F**). There is malacia (★) of the brainstem with gliosis **(G**), and discrete perivascular cuffing formed by lymphocytes and macrophages at the cortex (**H**). Haematoxylin & Eosin stain. Bar, A, E 50 µm; B 5 µm; C 100 µm; D,F–H 20 µm.

The principal histopathological findings are graphically summarized in Fig. 2. Ballooning degeneration of the transitional epithelium of the urinary bladder (100%; 43/43) and of the epithelium of the renal pelvis (95%; 41/43), villous atrophy (atrophic enteritis) of the small intestine (93%; 40/43), and interstitial pneumonia (84%; 36/43) were the predominant histopathological alterations observed. In addition, there was severe hyperplasia (involving 3 to 13 layers of epithelial cells) of the urothelium of the renal pelvis, ureters and urinary bladder (Fig. 3A). Furthermore, rare intracytoplasmic eosinophilic structures, measuring 6–7 µm, and suggestive of viral inclusion bodies of SVA were observed in areas of ballooning degeneration of the urinary bladder (Fig. 3B) and in neurons within areas of nonsuppurative meningoencephalitis.

Neurological disease: evidence of brain disease was observed in 46% (6/13) of the piglets with clinical manifestations suggestive of ETNL. These histopathological alterations varied from nonsuppurative meningoencephalitis (*n* = 3; Fig. 3C), cortical laminar necrosis (*n* = 3; Fig. 3D), nonsuppurative choroid plexitis (*n* = 3; Fig. 3E,F), gliosis and discrete cortical oedema (*n* = 3), malacia (*n* = 1; Fig. 3G), and areas of perivascular cuffing (*n* = 1, Fig. 3H).

Cutaneous lesions: the histopathological lesions observed in the skin of these piglets consisted of vesicular lesions that varied from dermal ulcers and/or crust formations. These alterations were observed at the coronary bands (35%; 15/43), snout (35%; 15/43), metacarpal (19%; 8/43), and hoof (9%; 4/43), and consisted predominantly of moderate, multifocal to coalescing necrotizing dermatitis. Necrotizing dermatitis with crusting formations was characterized by severe necrosis of keratocytes at the stratum spinosum of the epidermis with inflammatory influx composed of intact and degenerated neutrophils, rare lymphocytes, and some histiocytes. Moreover, at the skin adjacent to this region of necrotic dermatitis, there was orthokeratotic hyperkeratosis, intracellular oedema (severe, multifocal, hydropic degeneration, and multifocal, moderate ballooning degeneration), irregular epidermal hyperplasia with rete pegs formation, anastomoses, and mild acantholysis. In rare cases, it was possible to observe intact intradermal pustules, formed by mild parakeratotic hyperkeratosis with an underlying accumulation of degenerate neutrophils.

Oral cavity: consisted of lesions classified as necrotizing glossitis (16%; 7/43), gingivitis (14%; 6/43), and cheilitis (12%; 5/43). These lesions were characterized by focally extensive erosions and/or ulcerations with severe accumulations of intact and degenerate neutrophils. In addition, at the mucosa adjacent to areas of necrotizing glossitis, gingivitis or cheilitis, there was intracellular oedema (multifocal, moderate hydropic, and ballooning degeneration) of the superficial epithelium.

Lymphoid tissue: lesions were observed in the mesenteric lymph nodes and mucosa-associated lymphoid tissue. These alterations consisted of follicular lymphoid hyperplasia (53%; 23/43) and depletion (35%; 15/43) of mesenteric lymph nodes; follicular lymphoid hyperplasia (37%; 16/43) and depletion (44%; 19/43) of the tonsils; and follicular lymphoid hyperplasia of Peyer’s patch (28%; 12/43). The lymphoid tissue of the spleen was not investigated in this study, since it was not possible to differentiate between the red and white pulp due to the poorly developed organs in young piglets^19^.

Intestinal and hepatic disease: lesions observed in the small intestine consisted primarily of atrophic enteritis (93%; 40/43), followed by fusion of villi (35%; 15/43). In addition, there were necrosis (30%; 13/43) and vacuolization (12%; 5/43) of apical enterocytes in piglets with clinical manifestations of diarrhoea. Furthermore, there was random lymphoplasmacytic hepatitis (42%; 18/43).

### Immunohistochemistry findings associated with* SenecavirusA* in newborn piglets

**Figure 4:**
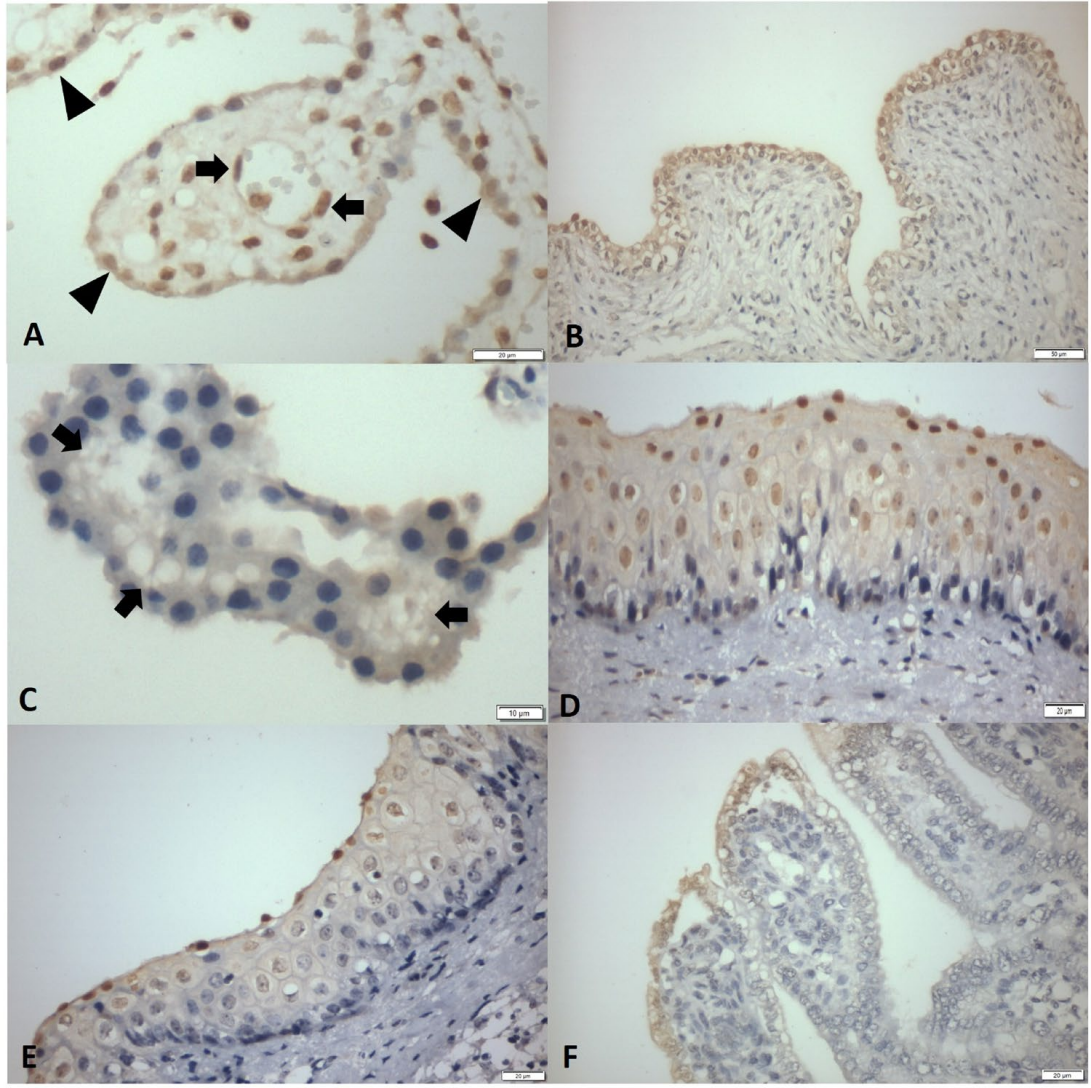
Immunohistochemical detection of antigens of SVA in epithelial tissues of newborn piglets. Cerebrum; there is positive immunostaining (arrow) at the capillary endothelium (**A**) and at the ependymal cells (arrow heads) of the choroid plexus (**C**). Urinary bladder; observe immunoreactivity to SVA at transitional epithelial cells (**B**). Observe the vacuolization of epithelial cells of the choroid plexus (**C**). Oral mucosa; observe positive immunolabelling of mucosal epithelium within areas of hydropic degeneration (**D,E**). Small intestine; there is positive immunolabelling of SVA at apical enterocytes within areas of intracytoplasmic vacuolization (**F**). Immunoperoxidase. Bar, A 50 µm; B,D-F 20 µm, and C 10 µm.

The principal tissues that demonstrated immunoreactivity to SVA were the urothelium of the urinary bladder (100%; 43/43), the renal pelvis (95%; 41/43), and the capillaries of the CP (Fig. 4A) of the cerebrum (81%; 35/43), with positive immunolabelling in areas of ballooning degeneration and epithelial hyperplasia of transitional epithelium (Fig. 4B). There was positive immunostaining of SVA at the epithelial cells of all piglets evaluated.

Furthermore, all piglets (# 1, 2, 3, 13, 26, 27) with neurological signs associated with ETNL and with histopathological evidence of brain disease demonstrated positive immunoreactivity to SVA at the CP and ependymal cells. In addition, there was severe vacuolization of epithelial cells of the CP (Fig. 4C) in all piglets with clinical manifestations of neurological disease.

There was positive immunoreactivity at the oral mucosa to SVA in 70% (30/43) of the piglets with severe hydropic degeneration (Fig. 4D,E), and positive immunolabelling for SVA in the apical enterocytes of the small intestinal in 63% (27/43) of all piglets with atrophic enteritis. Five piglets (# 4, 15, 17, 28, 38) presented intracytoplasmic vesicles at the apical enterocytes (Fig. 4F).

Furthermore, there was immunoreactivity to antigens of SVA in lesions at the coronary band (35%; 15/43), snout (35%; 15/43), metacarpal (19%; 8/43), tongue (16%; 7/43), gingiva (14%; 6/43), lips (12%; 5/43), and hoof (9%; 4/43). All pulmonary fragments (alveolar septum and respiratory epithelium) and lymphoid tissue of the tonsils evaluated were negative for SVA.

### Semithin and transmission electron microscopic findings

**Figure 5:**
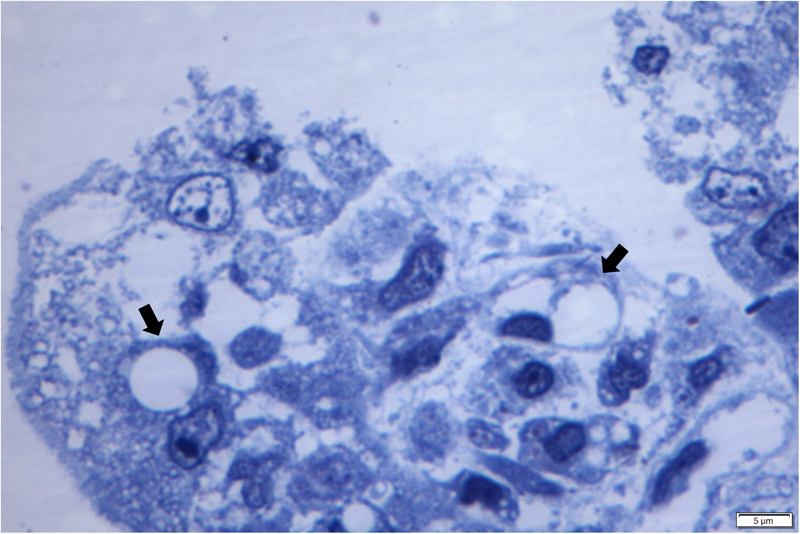
Semithin sections of the small intestine of piglets naturally infected with SVA. Observe SVA-induced balloon degeneration (open arrow) of the apical enterocyte of the small intestine resulting in displacement of the nucleus to the periphery. Bar, 5 µm.

Semithin (ST) evaluation through the apical region of the small intestine demonstrated severe vacuolization of apical enterocytes, with intracellular degeneration resulting in peripheral nuclear dislocation (Fig. 5). This virus-induced lesion must be differentiated from the physiological vacuolization of enterocytes associated with the ingestion of colostrum by piglets.

**Figure 6:**
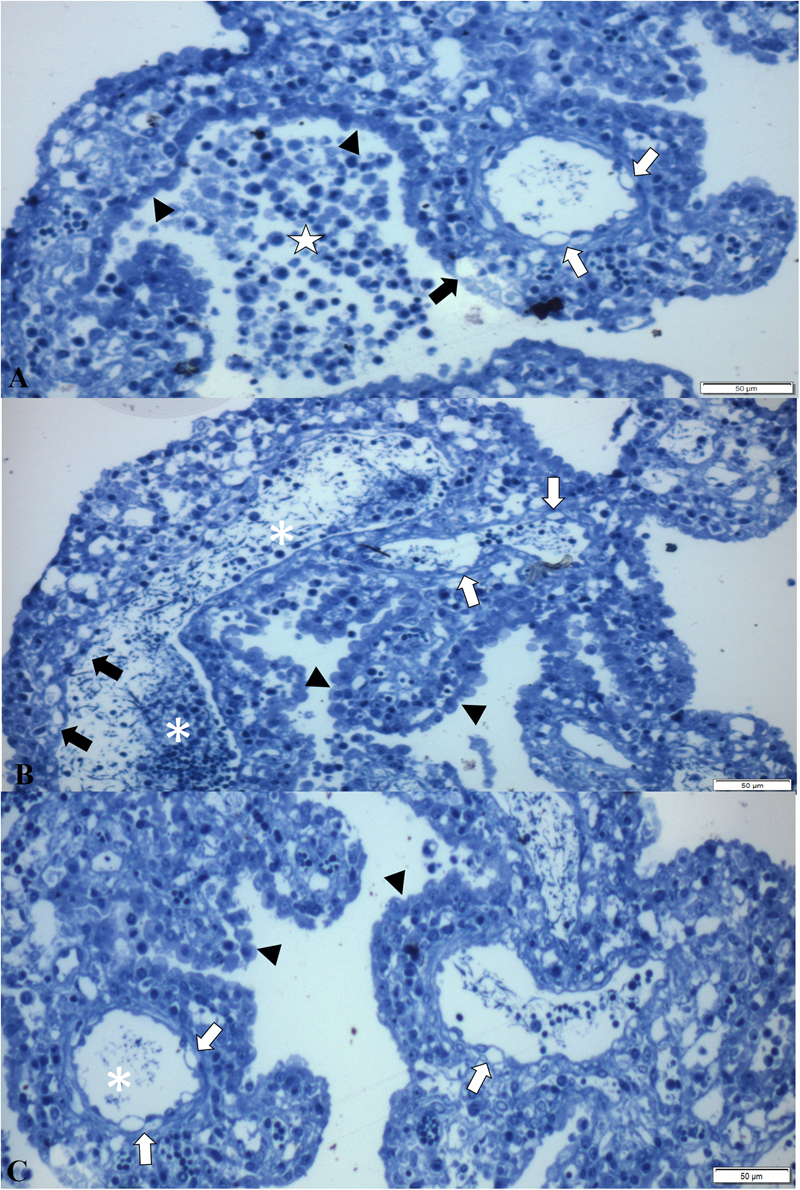
Semithin evaluation of the choroid plexus of piglets naturally infected with SVA. There is non-suppurative choroid plexitis (★), ballooning degeneration of endothelial cells (open arrows) of fenestrated capillary, degeneration (closed arrow) and swelling of (arrow heads) ependymocytes (**A**). Observe severe accumulations of macrophages and lymphocytes admixed with fibrin in the lumen (*) of a fenestrated capillary, necrosis (closed arrow) and degeneration (open arrows) of endothelial cells, with swelling and thickening (arrow heads) of ependymocytes (**B,C**). New methylene blue stain Bar, A-C, 50 µm.

Semithin sections of the CP revealed nonsuppurative choroid plexitis characterized by the accumulation of macrophages and lymphocytes admixed with fibrin within the ventricular lumen and fenestrated capillaries (Fig. 6A,B). In addition, there was marked ballooning degeneration and necrosis of endothelial cells of fenestrated capillaries with foci of swelling and degeneration of ependymal cells of the CP (Fig. 6B,C). Moreover, in some areas, the swollen ependymocytes resulted in discrete thickening of the ependymal layer (Fig. 6C).

**Figure 7:**
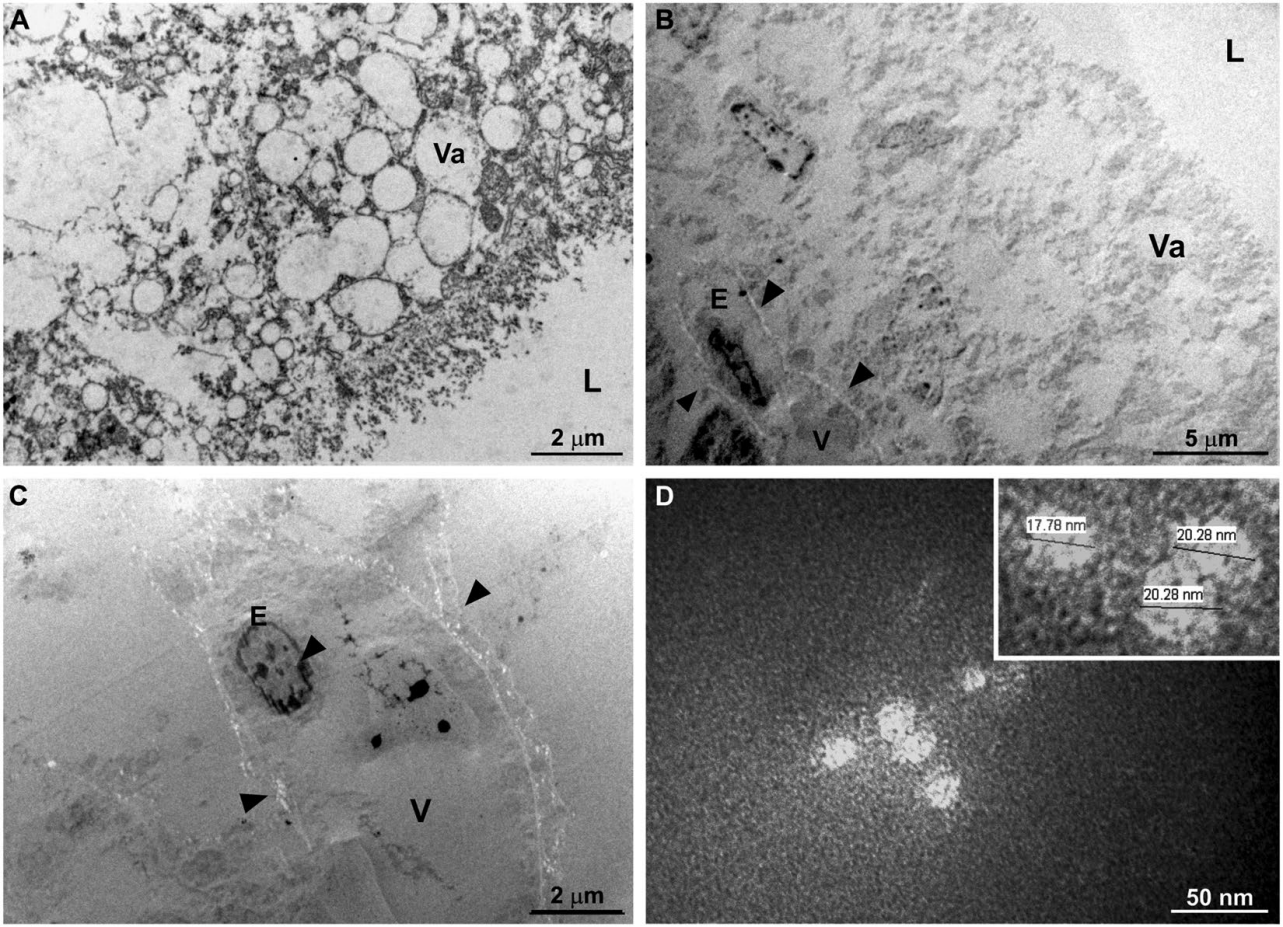
Transmission electron micrograph of the apical enterocytes of the small intestine. Electron microscopy of positive stained demonstrating that normal non-infected apical enterocytes of the small intestine contained numerous enlarged vacuoles (Va), intestinal lumen, L (**A**). Negative staining revealed that normal enterocytes had vacuoles (Va) of different sizes without viral particles, while infected cells had marked hydropic degeneration (vesicle, V) and viral particle aggregates surround (arrow heads) (**B,C**). Non-enveloped virus particles (**D**). Inset: high magnification of virus particles shown in (d) measuring 17 to 20 nm.

Transmission electron microscopy (TEM) of the choroid plexus and the apical region of the small intestine revealed information that confirmed the participation of SVA in the development of ETNL in newborn piglets despite the quality of the images which was affected by the method of collection (see below). Consequently, essential details of cell morphology were compromised and thus not observed. Nevertheless, TEM evaluation of positively stained sections of the small intestine demonstrated that normal apical enterocytes of newborn piglets have different-sized, enlarged macromolecules associated with the intestinal absorption of colostrum (Fig. 7A), which when negatively stained did not contain viral particles (Fig. 7B). However, infected apical enterocytes demonstrated hydropic degeneration and were surrounded by viral particle aggregates (Fig. 7 B,C); viral particles were observed only in apical enterocytes (Fig. 7D). Furthermore, there was swelling and rupture of the cisternae of the rough endoplasmic reticulum of infected apical enterocytes (data not shown).

**Figure 8:**
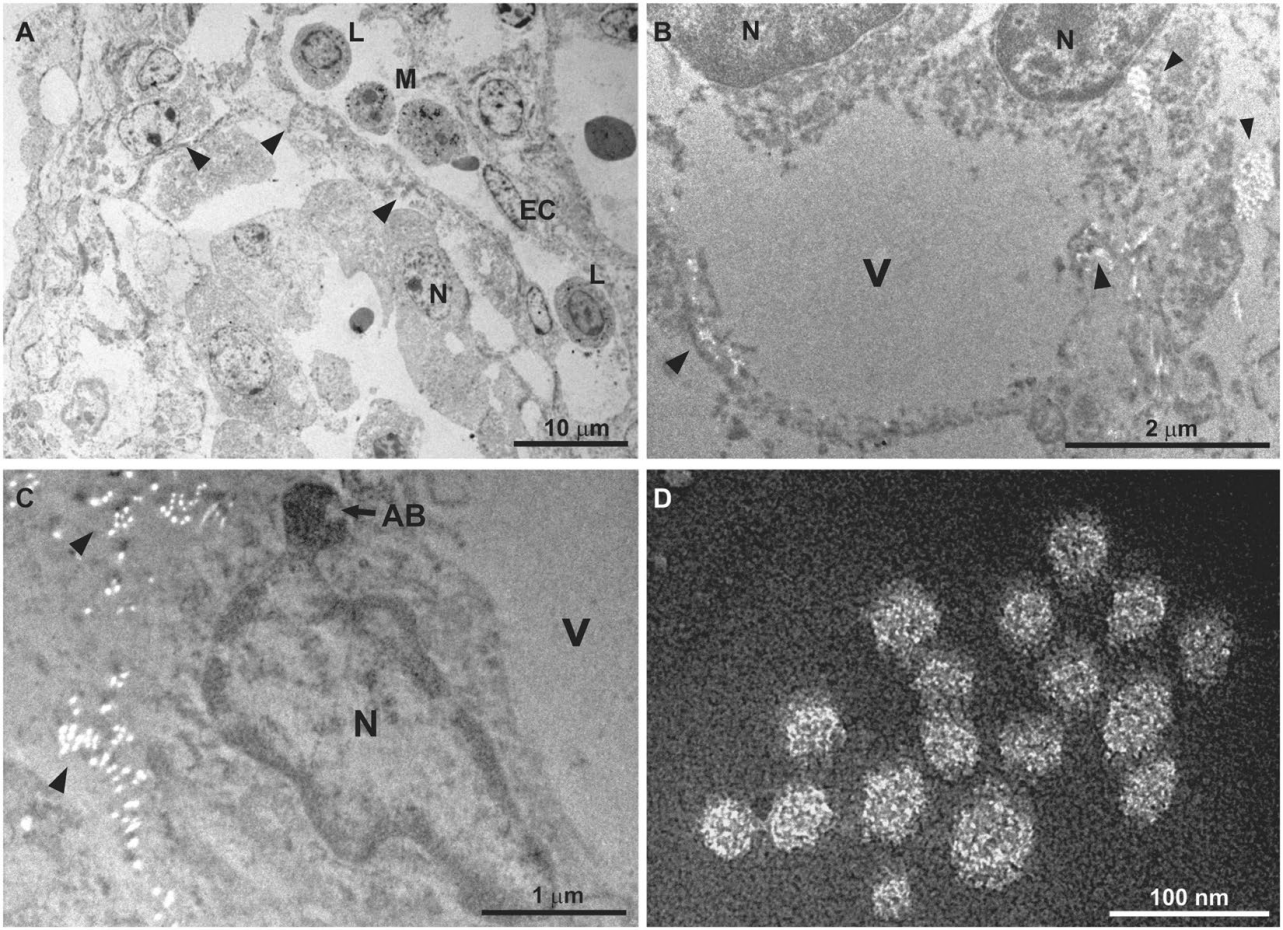
Transmission electron micrograph of the choroid plexus of newborn piglets naturally infected with SVA. Positive staining of the CP revealed the fenestrated capillary (arrow heads) containing an influx of lymphocytes (L) and monocytes (M); the tight junction (arrow heads) between adjacent endothelial cells (EC) is shown. N, nucleus (**A**). Observe the negatively stained balloon degeneration of ependymocytes characterized by intracytoplasmic vesicle (V) that is surrounded by aggregates (arrow heads) of SVA (**B**). There is an infected endothelial cell of the CP with an apoptotic body (AB), a vesicle (V) and aggregates of SVA (head arrow) (**C**). Closer demonstration of the non-enveloped virus observed in the cytoplasm of the infected endothelial cell of the CP (**D**).

Transmission electron microscopy of positively stained CP revealed an influx of lymphocytes and macrophages within the fenestrated capillaries of the CP, and that these leucocytes contained intracytoplasmic phagolysosomes (Fig. 8A); infected macrophages were not seen translocating via the tight junctions. Furthermore, negatively stained endothelial and ependymocytes infected by SVA demonstrated balloon degeneration, characterized as cytoplasmic vesicles (Fig. 8 B,C) surrounded by viral particles of 17–30 nm in diameter (Fig. 8D). In addition, in an endothelial cell of the CP, the nucleus was displaced to the margin of the cell, with detachment of the nuclear membranes, enlargement of the perinuclear space, deformation of the nuclei, and condensation and fragmentation into an apoptotic body (Fig. 8C). These findings are hallmarks of apoptosis suggesting that the balloon degeneration caused by SVA progressed to individual cell death.

The viral particles observed in the small intestine and CP were similar in size and morphology, demonstrating the pleomorphic non-enveloped viral particles of approximately 17–30 nm in diameter which is consistent with that of a picornavirus. The overall findings are compatible with SVA infection in these cells, and probably suggests an enteric-neurological dissemination pathway for SVA.

## Discussion

The results of the TEM and ST investigation have demonstrated that the vacuolization observed at the apical enterocytes of the small intestine during this and in a previous report of our group by histopathology^3^, were in fact viral induced, since there were degeneration and vacuolization of enterocytes, representing early manifestations of cellular injury. Similarly, the TEM and ST findings of degeneration and swelling of epithelial and endothelial cells of the CP (that were immunoreactive to SVA by IHC and contained viral RNA by RT-PCR) associated with intralesional viral particles confirmed the participation of SVA in the development of ETNL in these piglets.

An interesting finding during this investigation was the positive immunoreactivity of SVA to epithelial cells of the CP and ependymal cells of the lateral ventricle in six piglets with neurological manifestation associated with ETNL; similar the Haematoxylin & Eosin and IHC findings with combined RT-PCR amplification of SVA were previously described in newborn piglets^3^. SVA is a member of the family *Picornaviridae*, which includes several human and animal neurotropic enteroviruses (EV) such as porcine teschovirus (PTV)^20^, poliovirus, and enterovirus 71^21^, that produce diseases that begin in the gastrointestinal tract with subsequent neurological disorders, including nonsuppurative encephalomyelitis with perivascular cuffing^20^, aseptic meningitis^21^, and neuronal necrosis^20,21^.

In human pathology, it was postulated that viral dissemination of EV may occur via several mechanisms including disruption of the blood-brain-barrier, BBB^22^, and the blood-cerebrospinal fluid barrier, BCSFB, located between the cerebrospinal fluid and blood vessels of the CP^15,17^. Moreover, experimental studies have shown that PTV enters the brain hematogenously^20^. Furthermore, we have previously demonstrated antigens of SVA at the endothelial cells of capillaries within the CP^3^, which may indicate that SVA has the capacity to alter the permeability of the BCSFB in newborn piglets and probably result in nonsuppurative meningoencephalitis and cerebral cortical oedema with neuronal necrosis as herein described; cerebral oedema is a manifestation of dysfunction to the BBB^16,18^. Consequently, these theories are in accordance with the ST and TEM findings during this study which demonstrated degeneration and necrosis of endothelial cells of fenestrated capillaries of the CP and nonsuppurative choroid plexitis associated with intralesional viral particles. This then suggests that SVA produces an initial inflammatory response at the CP, enters the brain probably due to disruption of the BCSFB, and produces nonsuppurative meningoencephalitis and neuronal necrosis in newborn piglets. However, it is currently unknown what mechanism is used by SVA to cross the BCSFB. Infectious agents may cross the BCSFB via tight junctions between CP epithelial cells^16–18^, probably by infecting macrophages^17,18^, resulting in leucocytic trafficking. Alternatively, organisms may use the “Trojan horse” effect by infecting circulating leukocytes and then the brain parenchyma^23^, or colonize and infect epithelial cells of the CP before gaining entry to the brain^17^. Nevertheless, members of the *Picornaviridae* family are known to enter the brain via the BBB or neuromuscular junctions^23^. Although we were unable to observe infected leucocytes translocating through tight junctions of CP epithelial cells by TEM, this might be the most likely form of entry of SVA to the brain, and probably only observed in controlled experimental studies. Therefore, additional investigations are needed to confirm this hypothesis.

In newborn piglets^3^ and adult pigs^24^ SVA has been associated with gastrointestinal disease. In addition, the apical cells of the small intestine of newborn piglets with SVA-associated enteric disease have demonstrated histopathological evidence of injury, were immunoreactive to SVA by IHC and SVA RNA was identified by RT-PCR^3^. In the current study, atrophic enteritis was identified in 93% (40/43) of the piglets with gastrointestinal disease, and positive immunolabelling for SVA in the intestine of 63% (27/43) of these piglets. Consequently, we postulate that SVA may produce disease in newborn piglets by first affecting the intestine, after colonizing and replication in the tonsils^24^, with the possibility of subsequent dissemination to the brain by disruption of the BCSFB.

During this study, vacuolization of the villi of apical enterocytes of the small intestine was observed in five piglets (# 4, 15, 17, 28, 38) by histopathology; all lesions contained antigens of SVA by IHC and SVA was identified by RT-PCR. The results of the current study can now confirm that these lesions are in fact SVA-induced ballooning degeneration of enterocytes as demonstrated by ST and TEM, and represents an early phase of viral infection. We have described similar findings in the small intestine of piglets in which there was positive immunolabelling for SVA and viral detection by RT-PCR^3^. This is additional evidence in support of the enteric-neurological dissemination theory proposed for SVA. Nevertheless, more studies are needed to understand the mechanism involved with this degenerative lesion and the dissemination of SVA to the brain. However, caution must be taken to differentiate the SVA-associated apical degeneration of enterocytes with that observed in newborn piglets. In normal piglets, macromolecules of colostrum passively obtained from the sow is absorbed via enterocytes^25^, resulting in enlarged vacuoles that are enhanced by swelling of apical enterocytes^26^. However, TEM evaluation from this study has demonstrated that enterocytes infected by SVA are swollen due to intracytoplasmic vesicles surrounded by viral particles, while macromolecules of entreocytes derived from passively obtained colostrum did not demonstrate evidence of viral infection.

This is the first study that evaluated the combined pathological, immunohistochemical, molecular, and TEM findings associated with SVA-induced ETNL in a large population of piglets. The results from this investigation confirmed the findings of a previous study by our group that used relatively fewer piglets^3^, ratifies that the tissues recommended for the IHC detection of SVA antigens are the urothelium, brain with the CP, in addition to ulcerative lesions at the hooves, coronary band, lips or snout, and erosive lesions of the tongue. Furthermore, these findings demonstrate that piglets under 10 days of age can be infected by SVA and develop a multisystemic disease^3–6^. The ballooning degeneration observed in this study by histopathology and confirmed by ST and TEM evaluations, is one of the most characteristic histopathological features described in SVA-induced infections^2–4^, and represents initial invasion of the virus. The widespread immunolabelling of epithelial tissues observed in this study, and as was previously demonstrated^3^, suggests that SVA has tropism for epithelia.

There are few experimental studies inoculating SVA in finishing pigs^24,27^, weaned piglets^28^, but there is no experimental study with newborn piglets thus far. Nevertheless, experimental observations have shown that SVA induces a transient viremia in pigs, that is observed 3–10 days post inoculation, and coincides with the acute phase of this disease^24^. Interestingly, most of the piglets (81%; 35/43) from this study that died due to ETNL were between 2–5 days of age. This may suggest that the clinical manifestations of SVA are more intense in newborn piglets, resulting in sudden death^2,3,5,6,9^ due to the immature immune system of these animals at birth. The immune system of pigs is developed during the perinatal period and becomes mature when piglets are between 5 and 7 weeks of age^29^; consequently, neonatal piglets would then be more susceptible to infection which may explain the elevated incidence of sudden death in ETNL.

In summary, it is proposed that SVA infects the apical enterocytes produces atrophic enteritis with consequent alteration of cellular permeability which may result in diarrhoea, that is frequently observed in piglets with ETNL^3,5^. Alteration of cellular stability of apical enterocytes was demonstrated by semithin and ultrathin evaluations during this study. The virus probably then disseminates through lymphatic vessels, replicates in lymphoid organs (primarily the tonsil), and produces a transient viremia^24^. Evidence from this study demonstrated that SVA produces lesions at the BCSFB of the choroid plexus of newborn piglets, as demonstrated by IHC, ST and TEM, and may suggest that this virus enters the brain via the BCSFB, considering that this via is used by members of the *Picornaviridae* family^23^. The virus produces an initial inflammatory response at the CP which results in disruption of the BCSFB due to SVA-induced degeneration of endothelial and epithelial cells of the choroid plexus with subsequent encephalitic dissemination resulting in cerebrocortical necrosis and nonsuppurative encephalitis. However, addition studies are being carried out to confirm this hypothesis.

## Conclusions

The results from this investigation have demonstrated that ETNL is a multisystem disease of newborn piglets. It is proposed that SVA infects the apical enterocytes and then produces nonsuppurative meningoencephalitis and neuronal necrosis after possible disruption of the BCSFB at the choroid plexus. This would then suggest that SVA probably infects the brain in a manner that is similar to that of enteroviruses.

The gross, histopathological, molecular, and IHC findings of the 54 pigs analyzed corroborate with the findings of the study previously realized by our group with reduced number of piglets. We suggest that the gross alterations more frequently associated with SVA are ulcerative lesions at several anatomical locations (including the tongue, muzzle, coronary band, and hooves), which must be differentiated from those observed in other infectious diseases of pigs. There is evidence to indicate that the ballooning degeneration of the transitional epithelium is the most frequent histopathological lesion associated with SVA in newborn piglets and that IHC can be used to identify antigens of SVA in affected tissues. We recommend the collection of fragments of the urinary bladder, CP, renal pelvis, oral mucosa, and ulcerative lesions for histopathological and IHC diagnoses of SVA in newborn piglets.

## Methods

### Animals and gross pathology

During June 2015 to November 2016, 54 piglets, between 1 to 10 days of age, from 23 farms located in the South and Southeast regions of Brazil with presumptive diagnosis of SVA were submitted to the Laboratory of Animal Pathology, Universidade Estadual de Londrina, for diagnostic investigation. These piglets had reported clinical signs suggestive of ETNL, including diarrhoea, neurological manifestations, reduced weight gain, and sudden death. All autopsies were done soon after death; tissue fragments (brain, heart, lung, kidney with ureters, liver, mesenteric lymph node, spleen, small intestine, skin and/or tongue when injured, tonsil, and urinary bladder) were collected for histopathology. Caution was taken to include the brain with the CP. All fragments were immersed in 10% buffered formalin solution for 48 h and then routinely processed for histopathological evaluation with the Haematoxylin and Eosin (H&E) stain.

### Immunohistochemical identification of *Senecavirus A*

Selected FFPE tissue fragments of the brain with CP, lung, renal pelvis, small intestine, tonsil with oral mucosa, urinary bladder, skin, and tongue with erosive or ulcerative lesions were processed for IHC. These tissues were selected since they were constantly positive for SVA by RT-PCR in a previous study by our group^3^, where it was demonstrated that this virus has tropism for epithelial organs. Therefore, this study only presents the IHC findings since, other related viral agents known to produce similar lesions in piglets^2,3^ were not identified by RT-PCR assays in these piglets. In addition, multiple tissues from all 43 piglets were positive for SVA RNA by RT-PCR as previously described^3^.

Commercial silanized slides (StatLab, McKinney, TX, USA) containing FFPE tissue fragments were deparaffinized, hydrated in alcohol baths and subjected to antigen retrieval, using citrate buffer (pH 6.0) with the pressure cooker system (Electrolux Pressure Cooker PCC10, São Paulo, SP, Brazil) for 2 min. Subsequently, there was blocking of endogenous peroxidase with methanol and hydrogen peroxide (3%) for 25 min. The primary incubation was done with a monoclonal antibody (1:50 dilution), kindly provided by Dr. M. Yang, National Centre for Foreign Animal Disease, Manitoba, Canada^30^, with overnight incubation at 4 °C. Incubation with the secondary antibody (SuperPicture™ Polymer Detection kit; Invitrogen Corporation, Camarillo, CA, USA) was done in a humid chamber for 25 min at 25 °C, after which the chromogen, 3,3′-diaminobenzidine (DAB, Invitrogen^®^ Life Technologies, Frederick, MD, USA), was added for 3 min. Finally, all slides were counter-stained with Harris haematoxylin and assembled with a commercial resin. Positive (uroepithelium of the urinary bladder the piglets) and negative controls were used in all IHC assays, since the urothelium is the tissue of choice for the diagnosis of SVA by RT-PCR and IHC^3^.

### Ultrathin and transmission electron microscopic

Tissue fragments of the CP and small intestine of one piglet with nonsuppurative meningoencephalitis and vacuolization of apical enterocytes that were collected during autopsy for routine histopathological analysis and maintained in 70% alcohol were selected for TEM processing. This was done primarily to identify viral particles in these tissues, based on our previous findings in newborn piglets with ETNL^3^. These sections (measuring 1 mm^3^) were rehydrated in ethanol gradient (50–30° GL) and washed three times in 0,1 M PBS. The samples were fixed in 3% glutaraldehyde in 0.1% cacodylate buffer, post-fixed in 1% osmium tetroxide in 0,1 M cacodylate buffer, dehydrated in ethanol gradient and, after two baths in propylene oxide, embedded in Araldite resin (Electron Microscopy Sciences, Hatfield, PA, USA). Ultrathin sections (60 nm) were obtained by using a ultramicrotome (Leica Ultracut UCT, Solms, Hesse, Germany), transferred to 200 mesh grids, stained with 2% uranyl acetate and lead citrate (Reynold’s solution) or 3% phosphotungstic acid, then analyzed and photographed using a transmission electron microscope (FEI Tecnai G2, FEI Company, Hillsboro, OR, USA).

## Animal welfare issues

All methods used during this investigation were approved by and carried out in accordance with the guidelines and regulations of the Universidade Estadual de Londrina relative to the usage of animals submitted for autopsy. The owners of all animals used during this study gave consent for their usage in diagnostic and scientific activities.

## Electronic supplementary material

Supplementary Material
