# Supplementary material for: Histopathological, immunohistochemical, and ultrastructural evidence of spontaneous Senecavirus A-induced lesions at the choroid plexus of newborn piglets
Source: Sci Rep. 2017 Nov 29;7:16555. doi: 10.1038/s41598-017-16407-0 (PMC5707367; doi:10.1038/s41598-017-16407-0)
Supplement: Supplementary file 1 — Supplementary Material [file 41598_2017_16407_MOESM1_ESM.pdf]

Histopathological, immunohistochemical, and ultrastructural evidence of spontaneous *Senecavirus A*-induced lesions at the choroid plexus of newborn piglets

Thalita E. S. Oliveira[≠,€], Mariana M. Z. Michelazzo[≠], Thiago Fernandes[±], Admilton G. De Oliveira[±,£], Raquel A. Leme[¥,§], Alice F. Alfieri[¥,§], Amauri A. Alfieri[¥,§], Selwyn A. Headley[≠,€]

Supplemental Figure 1. Distribution of the age of piglets that died with clinical manifestations of Epidemic Transient Neonatal Losses. The period of intense viremia was between 2 and 5 days of life, as demonstrated in experimental studies[1] (Joshi et al. 2016)

1. Joshi, L. R. *et al.* Pathogenesis of *Senecavirus A* infection in finishing pigs. *J Gen Virol* **97**, 3267-3279 (2016).

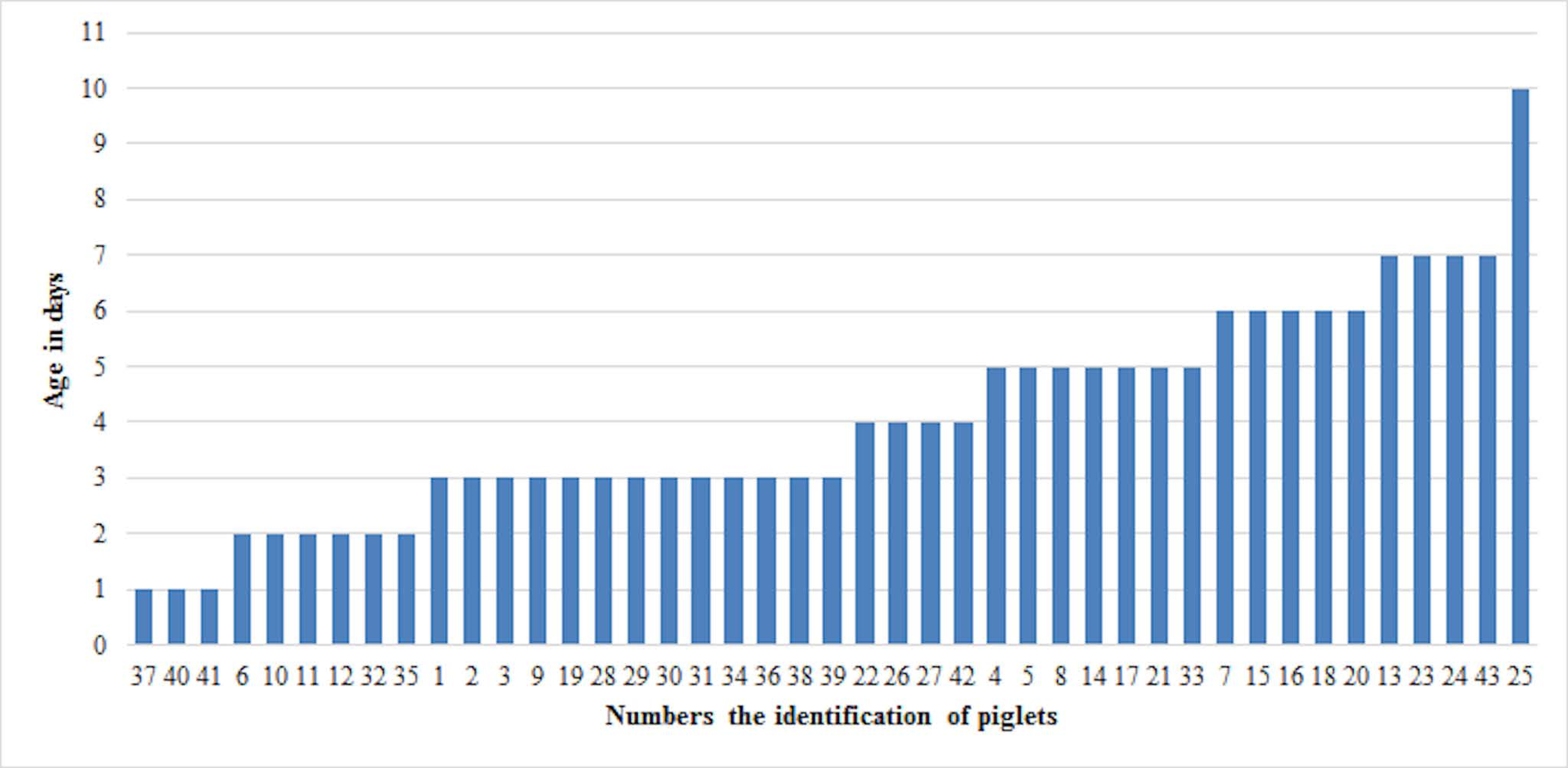
Age in days
11
10
9
8
7
6
5
4
3
2
1
0
37 40 41 6 10 11 12 32 35 1 2 3 9 19 28 29 30 31 34 36 38 39 22 26 27 42 4 5 8 14 17 21 33 7 15 16 18 20 13 23 24 43 25
Numbers the identification of piglets
